# Utilizing X-ray radiography for non-destructive assessment of paddy rice grain quality traits

**DOI:** 10.1186/s13007-025-01405-5

**Published:** 2025-07-09

**Authors:** Murugesan Tharanya, Debarati Chakraborty, Anand Pandravada, Raman Babu, Mahantesh Gangashetti, Swapna Paidi, Sunita Choudhary, Kaliamoorthy Sivasakthi, Krithika Anbazhagan, Bhavani Vaditandra, Michael Waininger, Mareike Weule, Eva Hufnagel, Joelle Claußen, Jiří Vaněk, Thomas Wittenberg, Jana Kholova, Stefan Gerth

**Affiliations:** 1 International Crops Research Institute for the Semi-Arid Tropics (ICRISAT), Patancheru, Telangana 502 324 India; 2 Multi-Crop Research Centre, Corteva Agriscience, Tunkikalsa, Wargal Mandal, Siddipet, Telangana 502336 India; 3 Development Center X-Ray Technology (EZRT), Fraunhofer Institute for Integrated Circuits IIS, Flugplatzstr. 75, 90768 Fuerth, Germany; 4 Department of Information Technologies, Faculty of Economics and Management, Czech University of Life Sciences Prague, Kamýcká 129, 165 00 Prague, Czech Republic

**Keywords:** Computer vision, Image data analysis, Quality control, Rice breeding, Rice phenotyping, X-ray imaging

## Abstract

**Background:**

Agricultural systems are under extreme pressure to meet the global food demand, hence necessitating faster crop improvement. Rapid evaluation of the crops using novel imaging technologies coupled with robust image analysis could accelerate crops research and improvement. This proof-of-concept study investigated the feasibility of using X-ray imaging for non-destructive evaluation of rice grain traits. By analyzing 2D X-ray images of paddy grains, we aimed to approximate their key physical Traits (T) important for rice production and breeding: (1) *T*_1_ chaffiness, (2) *T*_2_ chalky rice kernel percentage (CRK%), and (3) *T*_3_ head rice recovery percentage (HRR%). In the future, the integration of X-ray imaging and data analysis into the rice research and breeding process could accelerate the improvement of global agricultural productivity.

**Results:**

The study indicated, computer-vision based methods (X-ray image segmentation, features-based multi-linear models and thresholding) can predict the physical rice traits (chaffiness, CRK%, HRR%). We showed the feasibility to predict all three traits with reasonable accuracy (chaffiness: R^2^ = 0.9987, RMSE = 1.302; CRK%: R^2^ = 0.9397, RMSE = 8.91; HRR%: R^2^ = 0.7613, RMSE = 6.83) using X-ray radiography and image-based analytics via PCA based prediction models on individual grains.

**Conclusions:**

Our study demonstrated the feasibility to predict multiple key physical grain traits important in rice research and breeding (such as chaffiness, CRK%, and HRR%) from single 2D X-ray images of whole paddy grains. Such a non-destructive rice grain trait inference is expected to improve the robustness of paddy rice evaluation, as well as to reduce time and possibly costs for rice grain trait analysis. Furthermore, the described approach can also be transferred and adapted to other grain crops.

## Background

The shortage of food crops production is likely to escalate in the decades to come, facing the projected population growth and progressing climatic changes. The major staple food crops providing the base of the global diets and carbohydrates intake are cereals—namely maize, rice and wheat. For these crops, the yearly production improvement is marginal (< 1%; i.e. < 100 kg/ha [1, 2]). One of the important drivers of crops productivity in modern agriculture relies on crops research, that is expected to innovate the crop production methodologies to produce more food from less land and input. To achieve this, the agricultural research, particularly breeding, must significantly accelerate [3–5]. Despite of the rapid technological advancements, the accurate evaluation of thousands of potential new cultivars for the traits driving the marketable yield (e.g., related to success of the grain filling) and its quality (e.g., physical properties and biochemical composition of the grains) required in breeding process is still a challenge [6–10]. In the case of rice—as well as other crops having small and tightly husked kernels as, e.g., barley, oats, some millets, sunflower, or peanuts—the process of grain evaluation also includes the removal of husk (“de-husking”), which is not only time and cost intensive and destructive, but could be also the source of errors in evaluations [11, 12].

As witnessed in other disciplines, novel fit-for-purpose technologies can progressively bridge the gaps in knowledge and enhance the effectiveness of processes including agricultural research and crop improvement [13, 14]. For this, a wide array of non-destructive, sensor-based technologies are now available to evaluate various crop traits [15, 16]. These technologies have the potential to make the crops evaluation process more robust and improve its time and cost efficiency. In the case of rice breeding, three structural and physical grain traits are usually considered—e.g. grain chaffiness, chalky rice kernel percentage (CRK%) and head rice recovery percentage (HRR%). For each of these traits, different types of image-based technologies are being utilized e.g., near-infrared (NIR) sensors, standard RGB cameras, hyperspectral imaging, or nuclear magnetic resonance (NMR). Nonetheless, most of these imaging methods require the paddy rice to be de-husked and several different sensors need to be used [17]. Another challenge is that these image-based approaches typically generate a vast amount of spatial–temporal information (i.e., typically Terabytes of information in multiple dimensions), which must then be effectively analyzed. Therefore, one bottleneck to introduce such novel technologies in practical research is also linked to the challenges of high-volume data analysis and timely interpretation.

**Fig. 1:**
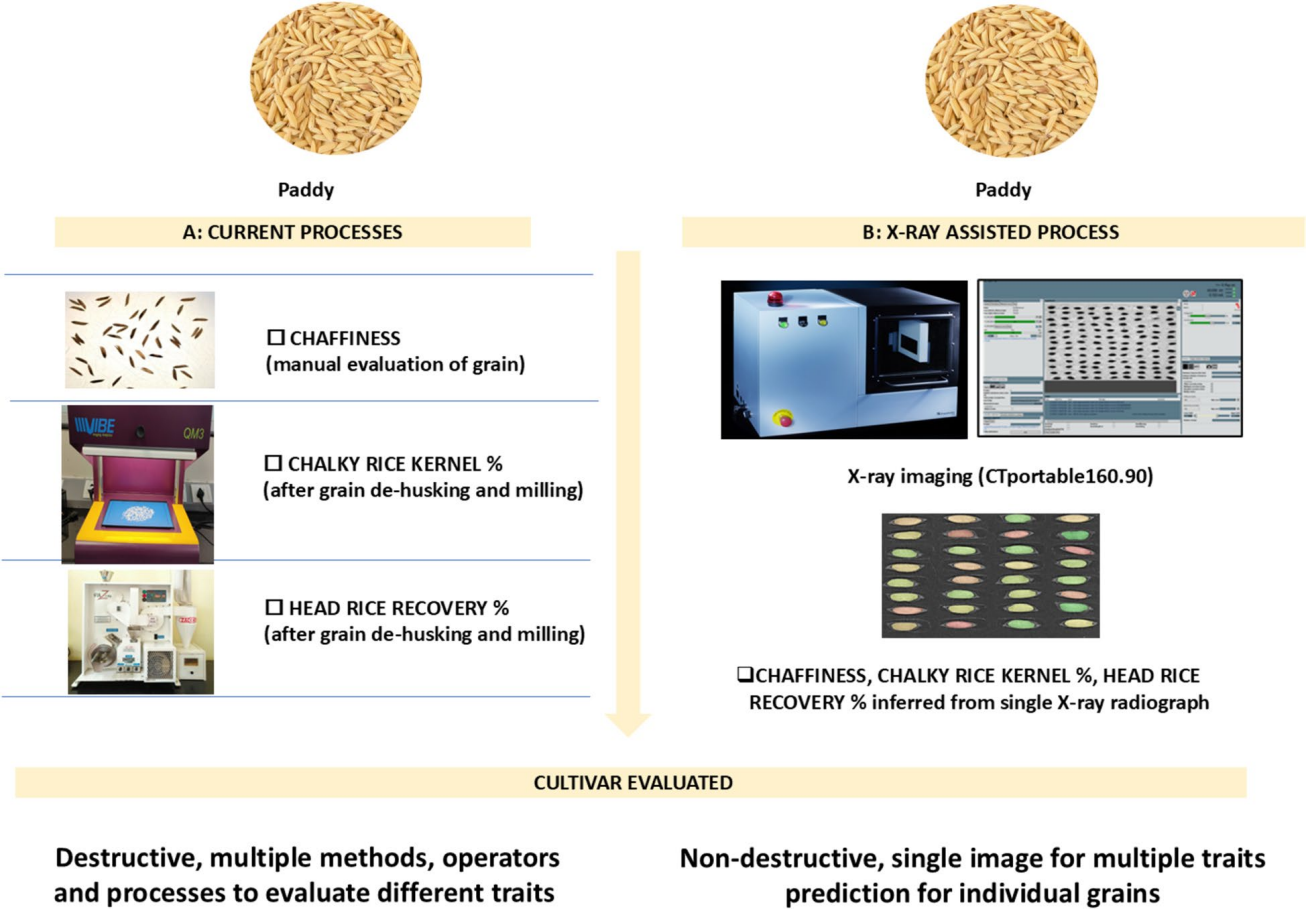
The left side of the figure (**A**) illustrates the methods typically required to evaluate rice grains (e.g. in rice breeding) for three rice grain properties “chaffiness”, “chalky rice kernel percentage” (CRK%), and “head rice recovery percentage” (HRR%). Whereas the right side of the figure (**B**) shows the steps involved and potential advantages if the proposed X-ray based solution would be used

Among the imaging technologies being already in use for rice-grain evaluation, X-ray imaging is rarely being discussed [18–22], although its advantages for the non-destructive evaluation of multiple physical rice grain properties as well as the evaluation of internal grain structure is evident [11]. In fact, it has been well-demonstrated with the new generation of high-resolution X-ray [23] or portable computed tomography (CT) systems (as e.g., the CT portable series system from the Fraunhofer EZRT^1^), that it is possible to skip the destructive part of grain evaluation for, e.g., in wheat [24] and peanuts [25]. Therefore, in this proof-of-concept study we will check whether the X-ray imaging technology has matured sufficiently enough to substitute and enhance key steps in rice grain evaluation. To demonstrate the approach, we have chosen three important physical grain traits (T) in rice breeding programs (visualized in Fig. 1):

(*T*_1_) “chaffiness”, the number of empty grains or grains with damaged or aborted embryos,

(*T*_2_) “chalky rice kernel percentage” (CRK%), kernels having a proportion of opaque, white, chalk‐like area and

(*T*_3_) “head rice recovery percentage” (HRR%), the percentage of unbroken kernel mass recovered after milling and polishing the grains.

## Materials and methods

**Fig. 2:**
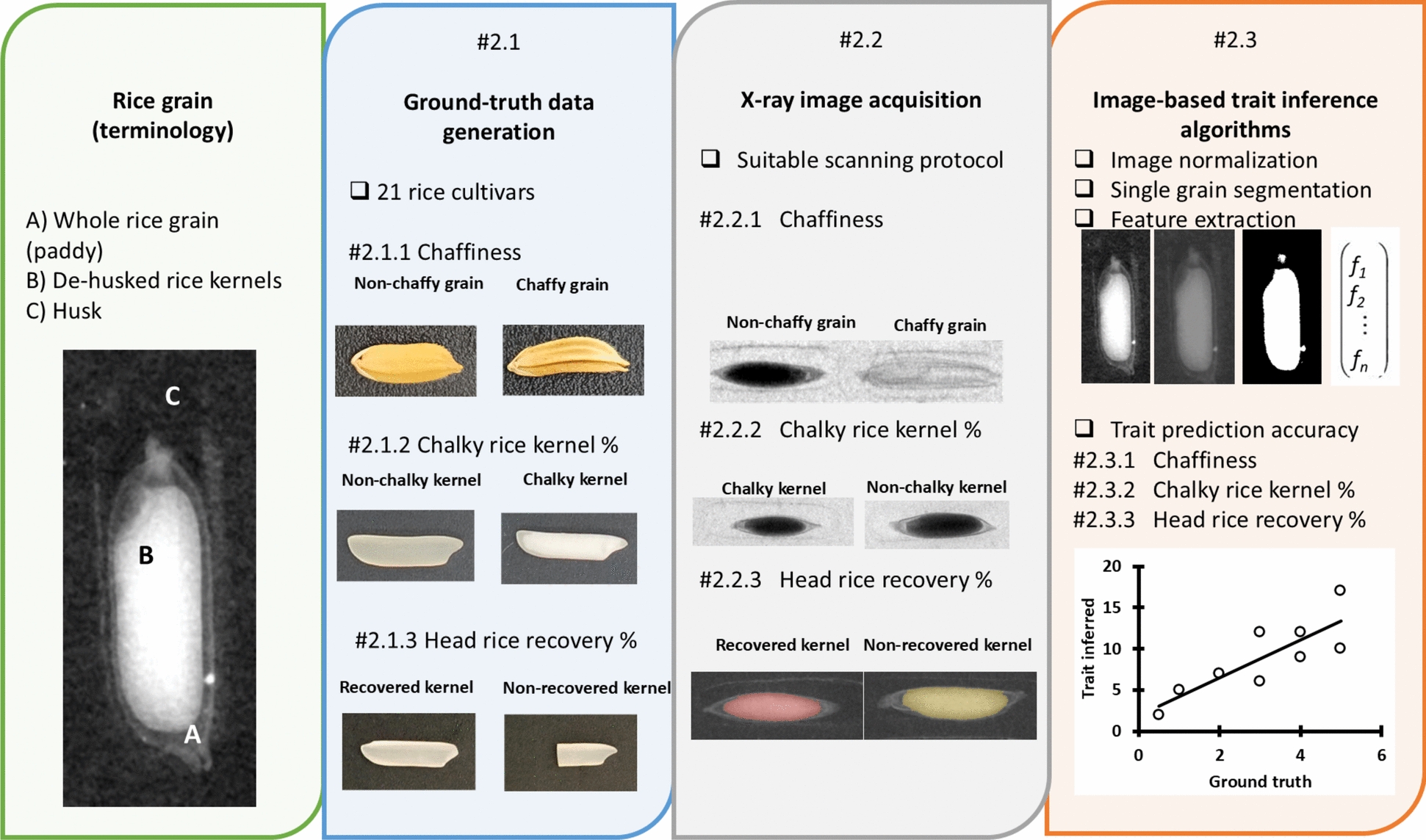
Overview of the methodological framework: The chart illustrates the logical sequence of steps (from left to right) followed in this study which are detailed in Sect. “Results”. From the left, these include description of the rice grain structure, diverse rice grain material and generation of ground truth measurements (Sect. “Rice grain material and ground truth measurements”), standardization of the paddy grain X-ray imaging process (Sect. “X-ray radiography”) and the image based structural and physical paddy grain traits inference (Sect. “Image treatment, features extraction and trait inference algorithms”)

The diverse rice grain material for the study and the ground truth measurements were generated using methodologies typically used for the paddy rice evaluation (Sect. “Rice grain material and ground truth measurements”). Section “X-ray radiography” describes the standardization of X-ray imaging process for paddy grain, while the structural and physical paddy grain properties inference from the X-ray images is detailed in Sect. “Image treatment, features extraction and trait inference algorithms”. Since the ground truth measurements, imaging and image analysis were different for each trait, individual sub-sections describe these procedures for each trait separately. Figure 2 illustrates the workflow of these consecutive steps organization of each section.

### Rice grain material and ground truth measurements

**Fig. 3:**
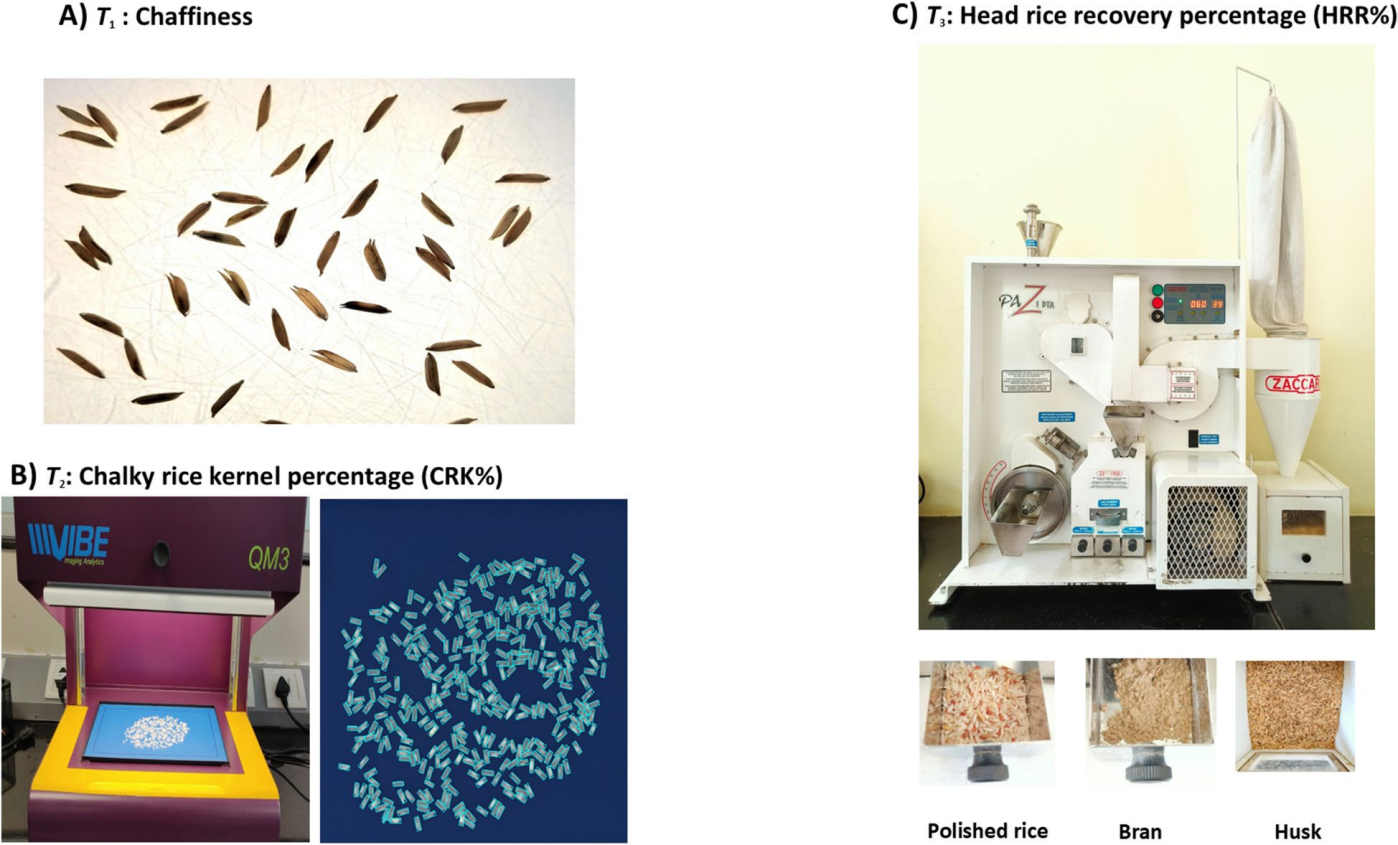
Different methods of acquiring the ground truth data for the three traits T_1_ Chaffiness—(**A**) manual counting using lightning board, T_2_ CRK%—(**B**) using VIBE scanner and software and T_3_ HRR%—(**C**) using Zaccaria milling machine. It should be noted that the T_2_ and T_3_ rely on de-husking and destructive analysis which had implications for generating inference algorithms for non-destructive X-ray based evaluation of whole grains

The rice cultivars’ selection was based on prior knowledge of their features and representing a sufficient range of the target traits variability. Altogether, 21 rice cultivars with different grain sizes and shapes were considered in the study: (a) long and slender; (b) short and bold (c) short and slender. Out of all cultivars, 6 were selected to assess chaffiness, 21 for CRK% and 9 cultivars for HRR%. All selected whole-grain samples (“paddy rice”) were imaged using 2D X-ray projections (Sect. “X-ray radiography”) and the ground truth was generated as per the protocols typically used in the breeding pipelines such as the one used in this proof-of-concept study (Fig. 3).

#### ***T***_***1***_***: Chaffiness***

“Chaffiness” (number of empty grains or aborted or damaged kernels) is the count of those grains which are either fully empty, the embryo has been aborted early in its development or damaged (e.g. by pests). Chaffiness indicates the success of pollination process and grain yield [12]. To generate ground-truth in our study, we spread the paddy grains on the lightning board and visually counted the chaffy grains (Fig. 3). The final chaffiness score (“chaffy”/“non-chaffy”) was based on the agreement of three different experts (method inspired by [26]).

#### ***T***_***2***_***: Chalky rice kernel percentage (CRK%)***

Chalky rice kernels can be visually identified after milling as those having a proportion of opaque, white, chalk‐like area in different parts of the kernel [27–29]. In our case, the ground truth CRK% measurements were estimated on de-husked kernels using an automated optical system (Vibe QM3 image analyzer, Vibe Imaging Analytics Ltd., USA, Fig. 3). This system evaluated each grain separately for the kernel translucence and considered the kernels with > 20% opaque area as chalky ones. Consequently, it calculated the proportion of chalky kernels in the sample (number of chalky kernels/total number of the kernels). Because the milling cannot be performed on a single grain level, in a first step, the single grains were first scanned, then manually de-husked, re-scanned, and the differences between these scans were used to adapt correlations with the X-ray images on un-husked rice paddy grains (Sect. “T1: Chaffiness”).

#### ***T***_***3***_***: Head rice recovery (HRR%)***

Head rice recovery percentage (HRR%) indicates the success of the industrial milling process, namely the recovery of polished rice kernels mass compared to the mass of raw paddy rice material. In our study, we used a milling machine (Zaccaria rice machine—Type PAZ-1-DTA, Zaccaria, Brazil; Fig. 3) which was specifically developed to evaluate HRR%. Since grain humidity does affect the milling process [30], all the samples were dried to moisture content of 12—14% before milling. The Zaccaria milling machine requires a minimum of 20 g of paddy rice, precise settings and a skilled operator for reliable measurements. In our case, the pre-weighted 20 g of paddy rice was milled and polished for 1.25 min. After milling, the fraction of polished grains (grains retaining more than 75% of the original length) was automatically separated by the machine and then manually inspected and cleaned. Afterwards, only the polished grains were weighted. The HRR% was calculated as follows:

$${\text{HRR}}\% = \, \left( {W_{{{\text{pg}}}} /W_{{{\text{op}}}} } \right) \, *{ 1}00,$$

where *W*_pg_ is the weight of the polished grains retaining more than 75% grain length and *W*_op_ is the weight of original paddy weight.

### X-ray radiography

All X-ray images were obtained using a micro-CT system (“CTportable160.90”, developed by the “Development Center X-Ray Technology” (EZRT) of the Fraunhofer Institute of Integrated Circuits IIS, Fürth, Germany). The system can be obtained commercially via system integrators (e.g. PhenoKey, NL). The micro-CT system consists of an X-ray source with acceleration voltages *U* from 30 to 90 kV, and acceleration currents *I* from 50 up to 160 µA. The X-ray detector has an active area of 2304 × 1300 pixels with a pixel size of 49.5 μm^2^ and allows to select the acquisition time *t* of each individual image between 100–1000 ms. The sample stage between the X-ray source and the detector can be positioned with a minimum focus object distance (FOD) of *f*_min_ = 16 mm and a maximum FOD of *f*_max_ = 285 mm, resulting in a maximum resolution of about 2.8 μm. The detector includes a 14-bit CMOS sensor (Teledyne DALSA Shad-o-Box 3 K HS) featuring a direct-contact Gd2O2S scintillator (Kodak Min-R 2190) foil. The detector was positioned at a focus detector distance (FDD) of *f*_*FDD*_ = *296 mm*. The system functionalities were controlled by the software Volex10 (Fraunhofer IIS, Germany^2^). More technical details of the X-ray imaging system can be found in [31].

In this initial proof-of-concept stage, rice grains were scanned in a structured way to ease the image processing. For this, sample holder was hand-crafted out of extruded polystyrene (eps; material with minimum attenuation factor, see the Figs. 6, 7, 8). Using such sample holder, more than 100 individual grains (~ 3–5 g) could be positioned inside the fixed grid in less than 5 min and consequently scanned in less than one minute. This procedure allowed us to inspect and evaluate properties of individual grains non-destructively. Several combinations of the scanning parameters (*U, I, t, f*_FOD_) were tuned to achieve images suitable for the features extractions for each trait separately (Table 1).

**Table 1:** Parameter settings of the X-ray system for target traits: chaffiness, CRK% and HRR%

Setting	Chaffiness (T_1_)	CRK% (T_2_)	HRR% (T_3_)
Voltage U [kV]	60	35	35
Current I [µA]	103	160	160
Acquisition time t [ms]	300	600	600
Focus object distance f_FOD_ [mm]	285	161	161

The experiments for Chaffiness were conducted with a suitable magnification (M) of approximately 1 (M = f_FOD_/f_FDD_). However, for the subsequent experiments on CRK% and HRR% we realized that the magnification and thus the resolution in the 2D X-ray radiographs was not sufficient and we increased the magnification to about 1.8 and realized a resolution of about 27 µm per pixel. Additionally, we lowered the voltage settings to 35 kV to increase the sensitivity and the absorption of X-ray photons in the grains. The throughput for the new images was about a factor of 2 smaller in the measurement time. However, the measurement time of 0,3 compared to 0,6 s per scan was neglectable compared to the times for sample change and sample preparation. This is because a general-purpose micro-CT setup was used for this feasibility study, the final definition of throughput will be determined in later stages of technological development with hardware components and sample positioning especially tuned and selected for this purpose.

With these tuned settings, we could generate the required X-ray images for CRK% and HRR%. In general, also Chaffiness can be analyzed with increased resolution and lowered voltage setting.

#### ***T***_***1***_***: Chaffiness***

**Fig. 4:**
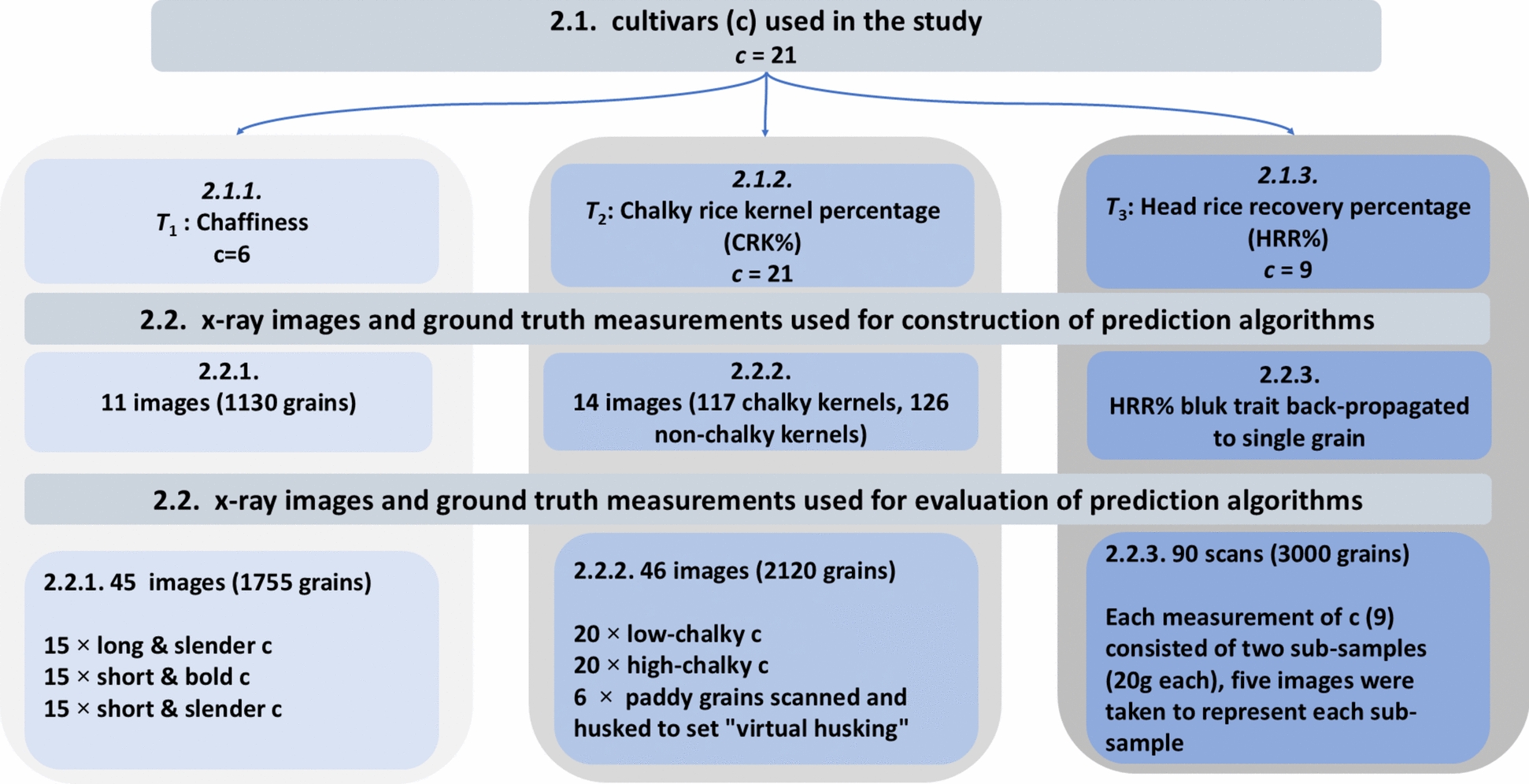
Overview of numbers of cultivars (c), X-ray images and grains used for the development of trait prediction algorithms for the three traits (T): T_1_ chaffiness (2.1.1), T_2_ chalky rice kernel % (CRK%, 2.1.2) and T_3_ head rice recovery % (HRR%,2.1.3)

The prediction algorithms were defined for 11 X-ray images annotated by experts. Consequently, 45 radiographs of real-situation grain samples were obtained (15 of each group: long and slender; short and bold and short and slender; altogether 1755 grains) for evaluation of prediction algorithms (Fig. 4). Figure 6A for X-ray images with rice grains depicted for chaffiness.

#### ***T***_***2***_***: Chalky rice kernel percentage (CRK%)***

To construct the CRK% prediction algorithms, it was necessary to define the chalky matter on de-husked kernels first. Since each kernel could include different proportions of chalky matter, we visually hand-picked the kernels with maximum/minimum chalky areas (fully translucent/fully opaque) in the representative rice kernel types (long/short, slender/bold) and consequently tuned the scanning protocol for de-husked chalky and non-chalky kernels to magnify the differences between these two classes (scanning parameters in Table 1). We used 14 different X-ray images (117 handpicked chalky and 126 non-chalky kernels) to develop the CRK% prediction algorithms (and Fig. 7B for X-ray images with rice grains picked for chalky rice kernel percentage). Additionally, paddy grains from 6 images were individually manually de-husked and re-imaged to validate the scanning parameters and the “virtual de-husking” algorithms (Sect. “T2: Chalky rice kernel percentage”). Consequently, leveraging our prior knowledge of cultivars (outlined in Sect. “Rice grain material and ground truth measurements”), we utilized 20 radiographs each of low-chalky cultivars (20 images) and high-chalky cultivars (20 images) to test the prediction algorithm (described in Sect. “T2: Chalky rice kernel percentage”). Altogether, employing this setup involved capturing 46 X-ray images (2 × 20 + 1 × 6) of paddy rice grains, totaling 2120 grains (Fig. 4).

#### ***T***_***3***_***: Head rice recovery percentage (HRR%)***

HRR% is an aggregate of several grain traits including the matter partitioning between husk and kernel, grain and kernel size and shape, grain fragility (inclusive of CRK% “factors” and grain breakages). Technically, single grain HRR% cannot be determined because the milling machine (Zaccaria, Sect. “T3: Head Rice Recovery (HRR%)”) requires minimum 20 g of grain to reliably resemble the milling process (i.e. a single ground truth point represents bulk of grains). As CRK% was expected to play the role in HRR%, the same scanning parameters as for CRK% were used for HRR% (listed in Table 1, also Fig. 8B for X-ray images obtained for head rice recovery percentage). For the HRR% analysis, altogether, 9 different cultivars were assessed (90 scans, 30 grains per scan, each cultivar measured 5 times, Fig. 4) for manual ground truth measurements. Each of these samples were split into two sub-samples (minimum 20 g each) and the ground-truth HRR% was destructively measured by the Zacharia mill for each subsample (Sect. “T3: Head rice recovery percentage”).

### Image treatment, features extraction and trait inference algorithms

**Fig. 5:**
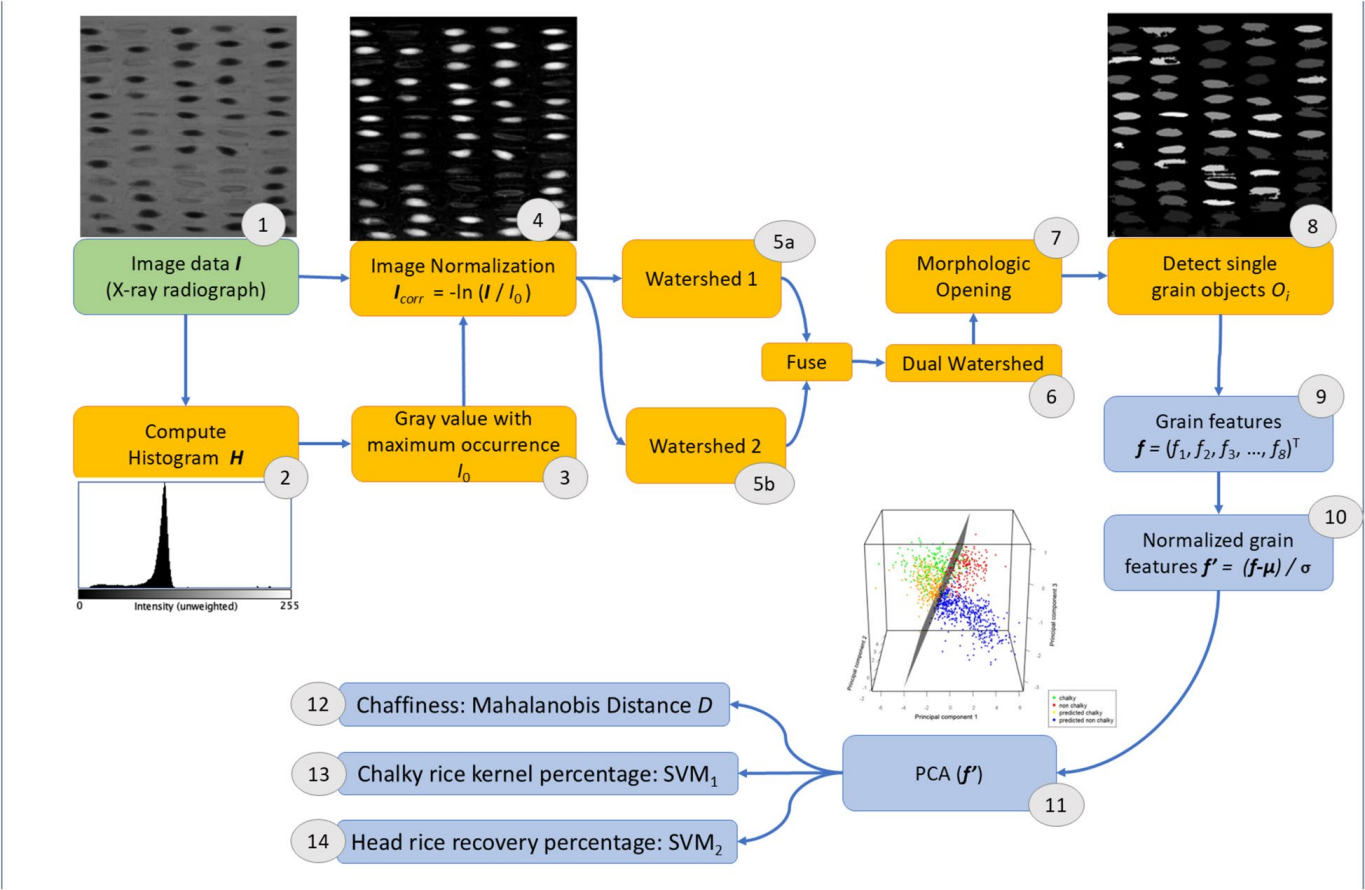
Workflow and image processing steps to extract grain features (9) for the PCA analysis (10) from X-ray images (1). The main steps include the computation of the histogram (2) and from that the identification of the gray value I_0_ with maximum occurrence (3). Combing I_0_ with the original image I, image normalization (4) can be achieved. Using a dual watershed approach (6) yields a ‘blob’ (= binary large object) for each grain. To eliminate small single pixels and fill some remaining holes in the blobs (noise), a morphological closing is applied to the blobs (7). Finally for each detected grain an individual image object O_i_ is identified (8). From each of these objects O_i_, a descriptive vector **f** = (f_1_, f_2_, f_3_, …, f_n_)^T^ is extracted (9) and normalized (10), which in turn are used to compute is the corresponding PCA model (11), the Mahalanobis Distance (12) and SVMs (13,14)

Each of the X-ray radiographs (Sect. “X-ray radiography”) was pre-processed in the following steps (Fig. 5):

1. **Image normalization** Fig. 5(4) was used to bring all acquired radiographs in the same grey-scale level range to be comparable to each other and at the same time correct for the exponential attenuation according to the equation for attenuation of Lambert–Beer. For normalization, the following equation is used:

$$I_{{{\mathbf{corr}}}} = \, - {\mathbf{ln}} \, \left( {I/I_{{\mathbf{o}}} } \right),A$$

where ***I***_**o**_ is the background intensity of the original X-ray image ***I*** Fig. 5(1). The value of ***I***_**o**_ is gained from the grey value histogram Fig. 5(2) of each individual X-ray image. Within this histogram the grey value with the highest number of occurrences is used for ***I***_**o**_ Fig. 5(3). Due to the design of the sample holder most of the area at the detector was not covered with individual seeds, thus the most prominent grey value in the histogram is the one corresponding to the unattenuated background intensity.

2. For **single grain segmentation** a variation commonly known as a blob-analysis [32] was made, consisting of a dual water shedding [33] approach Fig. 5(6) for individual rice grain detection as well as foreground and background separation, followed by a classical morphological erosion-dilation combination (sometimes referred in the literature as”opening”) [34] to remove single pixels due to noise Fig. 5(7).
3. After the individual areas/segments *S* that corresponded to rice grains were segmented from the image background Fig. 5(8), several image-based **features f** = (*f*_1_, …, *f*_8_)^T^ **(**being the descriptors of segmented regions S within the image) were computed for each segment of a rice grain separately Fig. 5(9):
4. *f*_1_ = Grain size: size of the segment *S* in pixels (which can be converted to mm^2^)
5. *f*_2_ = Mean value: Mean grey value of the pixels in the segment *S*
6. *f*_*3*_ = Weight: Virtual weight as a sum of normalized grey values within the segment *S*
7. *f*_*4*_ = Standard deviation: Standard deviation of the grey values of the segment *S*
8. *f*_5_ = Sphere radius: Radius of a circle, which has the same area as the segment *S*
9. *f*_6_ = Average weight: The average weight is a weight proxy multiplying the mean value with the sphere radius
10. *f*_*7*_ = Minimum covering circle (MVC) radius: Radius of the minimum covering circle which encloses the segment *S*
11. *f*_8_ = Sphere ratio/segment shape: This is the ratio between the sphere radius *f*_5_ and the sphere MCS ratio *f*_7_. The value is between 1 and 0. 1 relates to a circle-like shape, while 0 indicates the shape of an infinitive long ellipse.
12. For each of the three trait prediction models (chaffiness, CRK%, HRR%) a subset of the image-based segments’ features ***f*** was selected based on their correlation to the ground truth trait measurements to **build a grain trait prediction algorithm from X-ray image-based features (f**_***x***_**):** The impact of each feature (*f*_1_, …, *f*_8_)^T^ on the correlation with the ground truth measurements was analyzed with a Principal Component Analysis (PCA), Fig. 5(11). Prior to the PCA, all image-based features were normalized using the Z-Transform (***f***’ = (***f***—***µ)/σ***) to be in the same numerical range Fig. 5(10). Consequently, only the image-based features explaining the significant portion of variation in the target ground truth trait values were selected and out of these the multi-linear regression model to correlate grain trait from combinations of image-based features was built. This model helped to separate the image segments (that correspond to scanned rice grains) in the multi-dimensional PCA-space into individual classes based on the features of their X-ray image projections.

#### ***T***_***1***_***: Chaffiness***

**Fig. 6:**
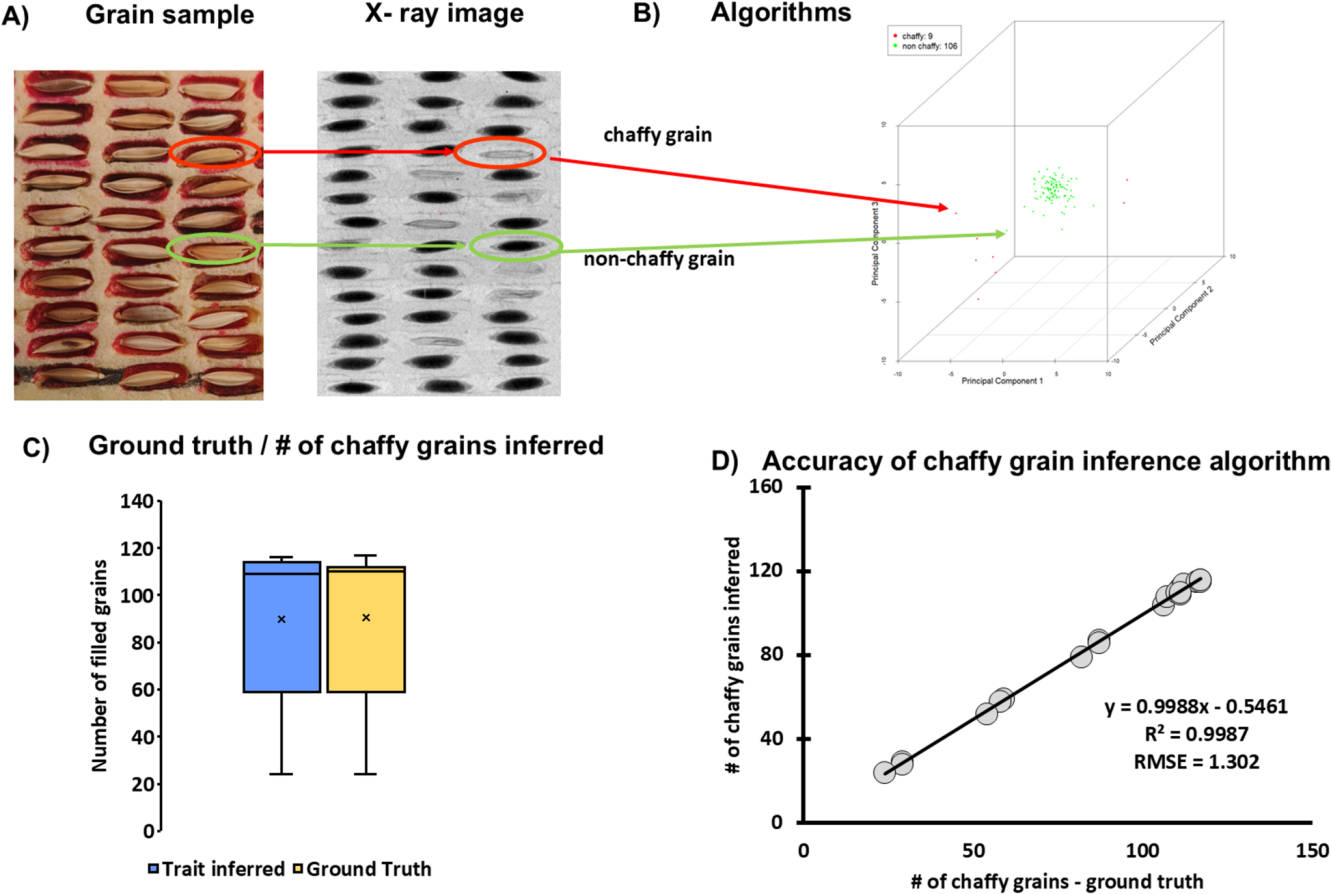
The key parts of the process required to build the”chaffiness” prediction model from the X-ray radiographs of paddy grains. **A** illustrates the sample in the sample holder and the raw X-ray image while pointing out the chaffy and non-chaffy grains we intended to predict (ellipses). **B** shows the distribution of features of “chaffy” and “non-chaffy” rice grains image segments in the three-dimensional PCA-space (PC dimensions 1, 2 and 3). Each of the dimensions in the plot represents a linear combination of the grain segments features (f_1_, …, f_8_)^T^ and the individual data points correspond to the image segments S that represent individual grain by the 3D coordinate in the respective dimension. The 3D plot also shows the results of the classification using a Mahalanobis-distance of 17 as threshold θ_chaffy_. The red data points are classified as “chaffy” and the green ones as “non chaffy” according to their distance to the point cluster. **C** shows the ranges of observed values estimated with standard methods (yellow) and the range of values predicted by the model (blue), (**D**) illustrates the agreement between the number of predicted and observed chaffy grains along with the standard goodness of the fit metrics (slope and intercept parameters of the linear regression, R^2^ and RMSE)

Rice chaffiness was defined as a binary property (Sect. “T1: Chaffiness”; each grain was considered as either chaffy or non-chaffy). Therefore, to separate grains identified as “chaffy” from “non-chaffy” we needed to find such principal components that could separate them similarly based on the combinations of features extracted from their images in PCA-space Fig. 5(11). For the PCA, all extracted grain features (*f*_1_, …, *f*_8_)^T^ were used as input. For further processing, the first three principal components (with a cumulated percentage of 99,974%) were used to discriminate “chaffy” and “non-chaffy” grains. These three PCAs were used to visualize the individual grains in a 3D plot Fig. 6B. Analysis of ground-truth data (Sect. “T1: Chaffiness”) showed that “non-chaffy” and “chaffy grains” formed defined clusters in the 3D PCA-space Fig. 6B. Thus, to separate the “chaffy” cluster from “non-chaffy” we had to find a threshold-distance, which described the border between the “chaffy” and the “non-chaffy” cluster. To achieve this, we used the Mahalanobis-distance [see Fig. 5(12)] which is an effective multivariate distance metric describing the distance between a point and a data distribution. The data distribution is characterized by a mean and the covariance matrix and is thus hypothesized to be a multivariate Gaussian Fig. 6 [35]. The threshold for this Mahalanobis distance was adjusted for the ground-truth scored by experts (Sect. “T1: Chaffiness”) to achieve maximum agreement of predicted values with ground truth observations (correlation metrics: coefficient of determination (R^2^), root mean squared error (RMSE)). The obtained threshold *θ*_chaffy_ in the normalized PCA space was *θ*_chaffy_ = 17 (arbitrary unit).

#### ***T***_***2***_***: Chalky rice kernel percentage***

The CRK% inference algorithms were initially built for kernels (de-husked grains). To determine CRK% from X-ray images, we considered CRK% as a binary property, i.e. rice kernel was classified as “chalky” if it contained more than 20% matter defined as “chalky”, and vice versa (details in Sect. “T2: Chalky rice kernel percentage (CRK%)”). Furthermore, we had to find suitable input parameters for the PCA Fig. 5(11) to separate the data points in the PCA-space. To this regard, a subset and combination of the following kernel image-based features were sufficient to explain the maximum variability in the ground truth observation: mean value (*f*_2_), standard deviation (*f*_4_), weight (*f*_3_) and averaged weight (*f*_6_) and were used as input parameters for the PCA. For analyzing the image-based kernel features in the PCA-space, the first three principal components were chosen. Furthermore, using the ground-truth estimates (Sect. “T2: Chalky rice kernel percentage (CRK%)”) it was possible to separate the PCA coordinates representing the “chalky” and “non-chalky” kernel images with a hyperplane. The hyperplane was calculated by a support vector machine [SVM, Fig. 5(13)] to achieve maximum agreement of inferred values with ground truth observations [correlation metrics: coefficient of determination (R^2^), root mean squared error (RMSE)]. In the next step, the ground truth rice grain measurements were compared with the image-based predictions to optimize the classification hyperplane. Since the chalky kernel matter had to be finally defined on whole rice grain including husk, the consecutive prediction algorithms had to be adjusted for estimation of CRK% from the radiographs of whole grains *with* husk. This adjustment has caused a shift in the data points’ coordinates in the PCA-space. To compensate for this shift, the “**virtual de-husking**” was introduced to adapt the PCA-parameters of each rice grain. To achieve this, first the mean differences between the PCA-parameter values of the ground-truth for kernels (without husk) and of grains (with husk) were calculated. This variation was used to normalize the input parameters by subtracting the values from the grain data with husk for each grain before doing the PCA and SVM classification:

$$p_{{{\text{PCA}},{\text{ kernel }} = }} {\text{p}}_{{{\text{PCA}},{\text{ grain}}}} {-} \, \left( {\mu \left( {p_{{{\text{PCA}},{\text{ GT}}}} } \right) \, {-}\mu \left( {p_{{{\text{PCA}},{\text{ grain}}}} } \right)} \right)$$

where *p*_PCA, kernel_ represents the principal component analysis (PCA) value for rice kernel (de-husked grain), *p*_PCA, grain_ denotes the PCA value for whole rice grain (with husk), *µ* (p _PCA, GT_) gives the mean PCA value for the ground truth, and *µ* (p _PCA, grain_) expresses the mean PCA value for whole rice grain (with husk).

**Fig. 7:**
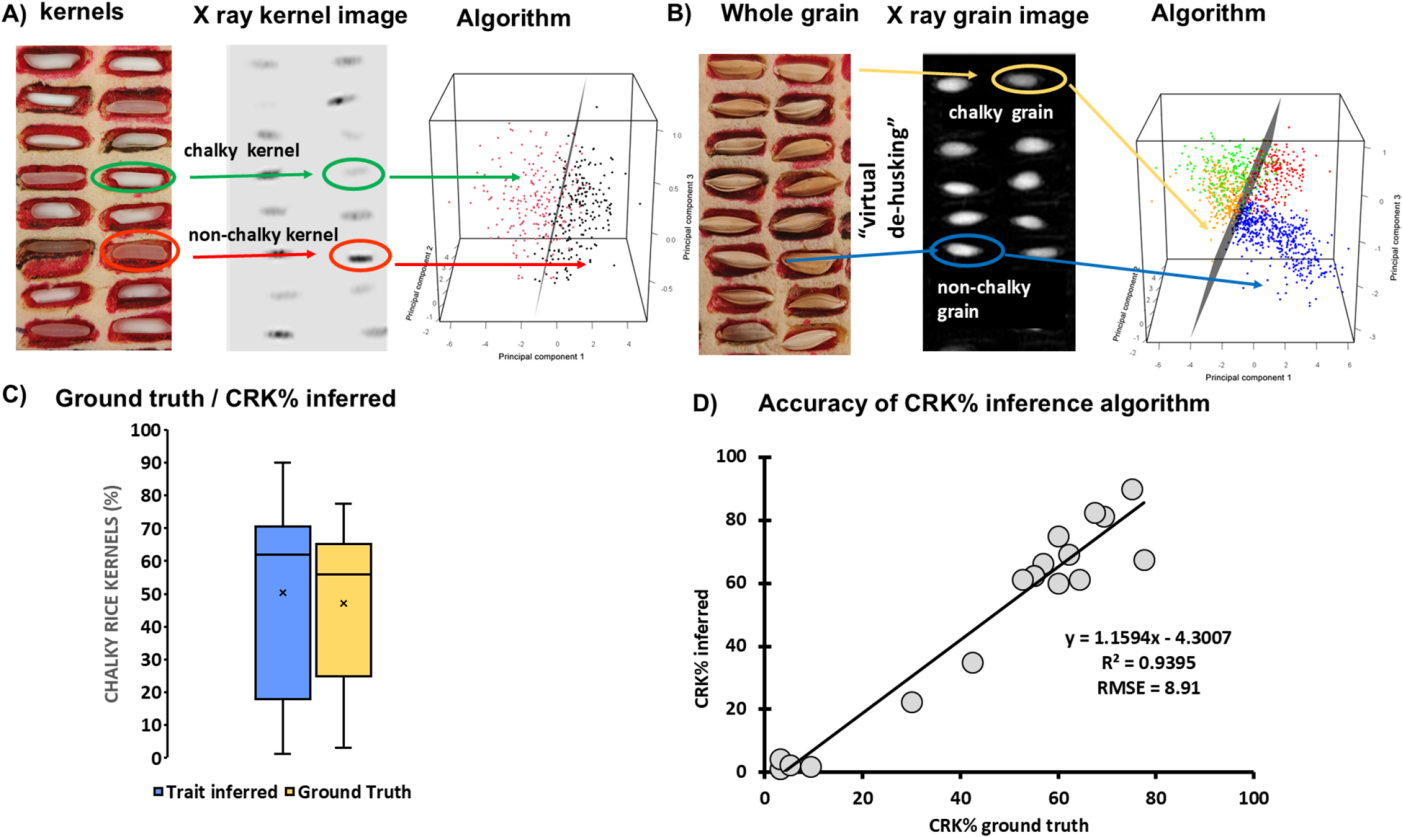
The key parts of the process required to build the” CRK%” prediction model from the X-ray radiography of paddy grains. **A**, **B** visualizes the sample holder with chalky and non-chalky kernels (**A**) and whole grains (**B**) and corresponding sample in the raw X-ray image-sections,with ellipses pointing out the type of grains we intended to predict. The graphs in A and B points out the data distribution of “chalky” (green, orange) and “non-chalky” (red, blue) rice kernels (**A**) and grains (**B**) in the 3D PCA-space. The dimensions are the three principal components (PCs), which are linear combinations of the PCA input parameters f2, f3, f4, and f6. The depicted hyperplane (gray) is the optimum to divide between “chalky” and “non-chalky” kernels and is calculated by a support vector machine (SVM). (**C**) shows the ranges of observed values estimated with standard methods (yellow) and the range of values predicted by the model (blue) while (**D**) illustrates the agreement between predicted and observed values along with the standard goodness of the fit metrics (slope and intercept parameters of the linear regression, R^2^ and RMSE)

This “virtual de-husking” shifted the data points in the PCA-space and enabled the classification of the complete grains (“with husk”) using the prior determined SVM plane (Fig. 7).

#### ***T***_***3***_***: Head rice recovery percentage***

Head rice recovery percentage (HRR%) was defined as a continuous property, which represented the ratio of unbroken head rice kernels recovered after grain milling. Unlike chaffiness and CRK%, HRR% ground truth cannot be estimated for individual grain. Thus it was not possible to directly associate each grain to a cluster in the PCA-space based on its features as it was done for chaffiness and CRK%. First, we separated individual ground-truth measurements for each grain subset (i.e. 20 g of grains, Zaccaria milling method, Sect. “T3: Head rice recovery percentage (HRR%)”). into five classes for the HRR%: “100% HRR%”, “80% HRR%”, “60% HRR%”, “40%” HRR% and “20% HRR%”. Consequently, we extracted and clustered the features (*f*_*1*_*, f*_*3*_*, f*_*4*_*, f*_*5*_ and *f*_*7*_) of the individual scanned grains in the PCA space Fig. 5(11). Then, based on these features, we fitted the SVM plane [see Fig. 5(14)] that allowed to assign each individual grain such probability of belonging to the HRR% class that achieved maximum agreement with average of all grains used to generate that particular single HRR% ground truth point [correlation metrics: coefficient of determination (R^2^), root mean squared error (RMSE)]. Then, the probability (*p*) of the individual grain belonging to a particular HRR% class is predicted, e.g. *p*_1_ = 21% for “100% HRR%”, *p*_2_ = 18% for “80% HRR%”, *p*_3_ = 66% for “60% HRR%”, *p*_4_ = 75% for “40% HRR%”, and *p*_5_ = 35% for “20% HRR%”. The most probable class max (*p*_1_, *p*_2_, *p*_3_, *p*_4_, *p*_5_*)* is then selected to predict HRR% of each individual grain. In this example the prediction would be *p* = “40% HRR%” with a reliability of prediction of 75%. This means that this grain has the most likely HRR% of 40%. Doing so we were able to classify HRR% for each individual grain based on the ground truth values of a grain subset.

## Results

**Fig. 8:**
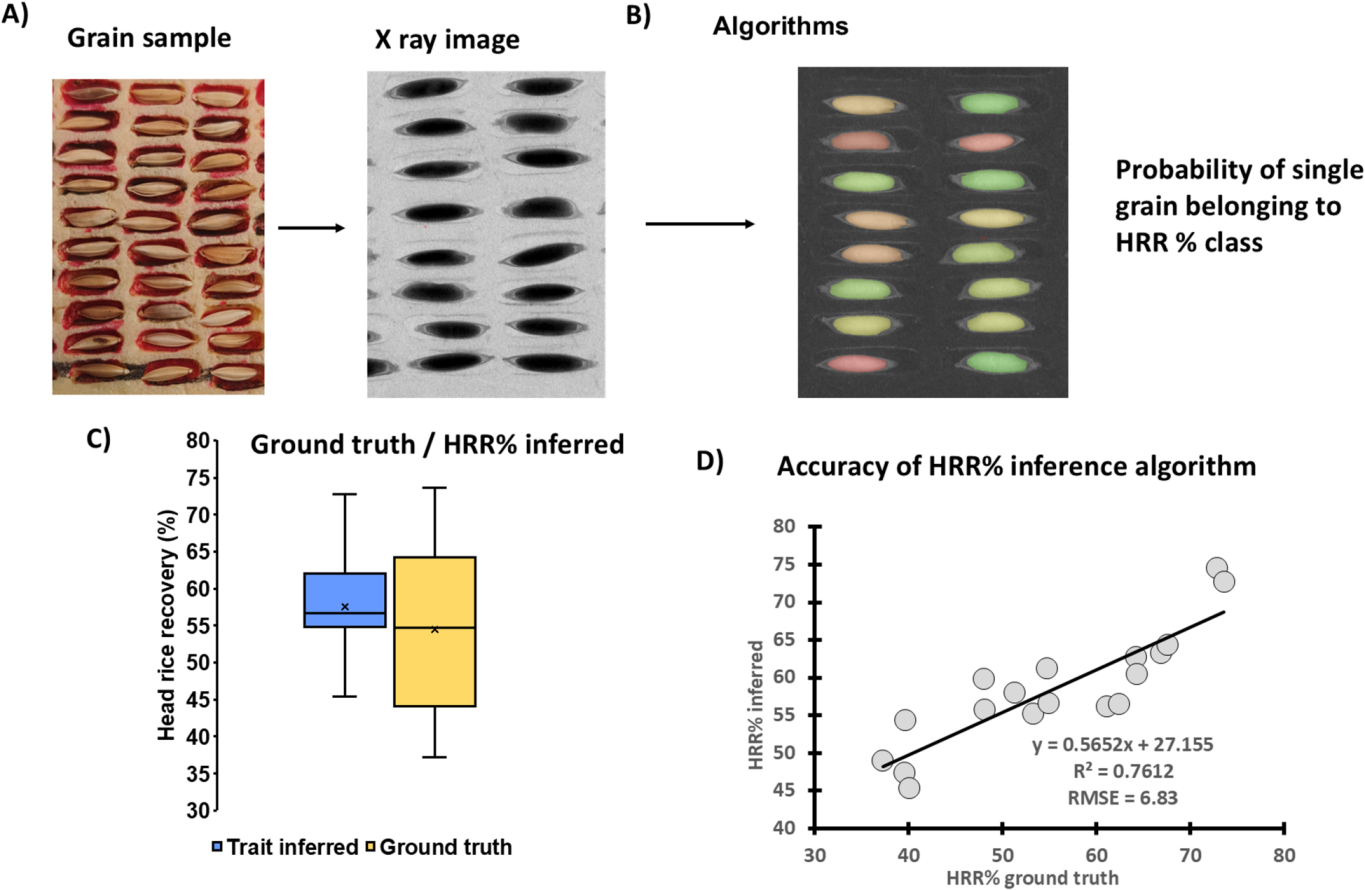
The key parts of the process required to build the “head rice recovery percentage” (HRR%) prediction model from the X-ray radiographies of paddy grains. **A** visualizes the sample holder and an example of raw X-ray image. **B** illustrates the HRR% classification algorithm with the colors representing the probability of each grain belonging to a particular HRR% class. **C** shows the ranges of observed values estimated with standard methods and the range of values predicted by the model. **D** illustrates the agreement between predicted and observed values along with the standard goodness of the fit metrics (slope and intercept parameters of the linear regression, R^2^ and RMSE)

21 diverse rice cultivars were selected to assess variability in three traits *T*_1_ = chaffiness (Sect. “T1: Chaffiness”), *T*_2_ = CRK% (Sect. “T2: Chalky rice kernel percentage (CRK%)”) and *T*_3_ = HRR% (Sect. “T3: Head rice recovery percentage (HRR%)”). We established an X-ray imaging set-up and an adequate scanning protocol to infer grain traits from the X-ray images of the grains. We manufactured grain sample holders (Fig. 6A) to hold ~ 100 rice paddy grains (~ 3–5 g) at a time. This sample holder needed approximately 5 min to be filled and around 1 min to be placed in the X-ray system, scanned and removed from the X-ray system. The selected whole-grain samples (“paddy rice”) were imaged using X-ray system (Figs. 6b, 7b, 8b) and, for the imaged samples, the ground truth was generated (Figs. 6a, 7a, 8a). The scanning procedure, the variability in the target trait (Figs. 6c, 7c, 8c), the key principles of features extraction process from X-ray images and the accuracy of the individual grain trait inference from these images (Figs. 6d, 7d, 8d) are described in the following sub-sections.

### ***T***_1_: Chaffiness (number of empty/aborted/damaged grains)

To establish the classification algorithm that differentiated “chaffy” grains, we used the X-ray images which were manually annotated by experts (Sect. “Image treatment, features extraction and trait inference algorithms”). The whole process is illustrated by Fig. 5. Section “Image treatment, features extraction and trait inference algorithms” the developed image analysis was able to differentiate “chaffy” and “non-chaffy” grains and reflected the scoring by experts with high accuracy (R^2^ = 0.9987, RMSE = 1.302; Fig. 6D).

### ***T***_2_: Chalky rice kernel percentage (CRK%)

To capture the variability in CRK% of paddy rice using an X-ray system, the image features defining chalky kernel mass had to be found. Therefore, unlike for “chaffiness”, the prediction of “CRK%” from X-ray images required a “virtual de-husking” step in the image analysis procedure that separated the proportion of image reflecting the husk mass from the kernel mass at the level of individual grains. The key sequence of the whole process is illustrated in Fig. 7. We observed that for certain grain types, particularly the short and bold grains, the generic “virtual de-husking” approach did not achieve a complete husk segmentation in the 2D X-ray images, hence leading to an overestimation of CRK% in these specific samples (four of low CRK% samples within our dataset predicted 60–70% CRK). However, for the remaining samples, the obtained CRK% predictions reflected substantial proportion of variation in ground truth CRK% (R^2^ = 0.9395, RMSE = 8.91).

### ***T***_3_: Head rice recovery percentage

During the algorithm development process, it became evident that HRR% is a complex trait influenced by multiple grain features, including the distribution of matter between the husk and kernel, grain and kernel size and shape, and grain matter homogeneity. In our dataset, the combination of “grain size” and “shape” image features, along with the features used to predict CRK% (kernel matter density and homogeneity within the kernel) were used to explain HRR% variability at the level of individual grains. **Error! Reference source not found.**igure 8 provides a visual representation of the steps involved in the process. It should be noted that the estimated HRR% predicted from the X-ray image analysis method overestimated the ground truth measurements (37–74% HRR, mean = 56% HRR), compared to estimations using X-ray image analysis (45–73% HRR, mean = 59% HRR). However, the predicted values were correlated with the manual measurements reasonably well (R^2^ = 0.7612, RMSE = 6.83), although the slope and intercept deviated from a 1:1 ratio.

## Discussion

Technological advancement of the agricultural sector is expected to address the food requirements of the growing population. The presented case study is the first of its kind that shows feasibility of using X-ray-based imaging and image analysis methods to assist rice research, particularly rice breeding process.

In our proof-of-concept study, we focused on three physical grain traits related to the rice crop yield and quality; *T*_1_ = number of chaffy grains (“chaffiness”, indicating the degree of grain filling or damage), *T*_2_ = chalky grain chalkiness percentage (CRK%, reflecting the kernel quality) and *T*_*3*_ = head rice recovery percentage (HRR%, indicating the amount of marketable yield after the paddy rice milling and polishing process). Currently, in most of the cases, the number of “chaffy” grains is counted manually while other traits like HRR% and CRK% require the grains to be first de-husked and polished mechanically (e.g. by milling machine), broken kernels need to be separated, and for HRR% the individual fractions have to be additionally weighted. These separated kernels undergo further evaluation for the proportion of chalky kernels with different machines (e.g., like Vibo) and/or visually.

These three key traits are the physical/structural properties of the grain which could be potentially measured using a single sensor like X-ray imaging [24] in combination with adequate image postprocessing. Such an approach has not been attempted for rice grains evaluation before. Furthermore, as recently shown for wheat [31] and peanuts [25], the trait calculation from X-ray images is non-destructive, and these images capture the grain mass variability along with its internal structures. This is a considerable advantage over other sensory methods like RGB or NIR imaging that typically capture only fraction of the grain surface. This particular property of X-ray imaging—being able to evaluate grain internal mass and structures (e.g. kernel and husk)—was leveraged for paddy rice in this feasibility study.

Among the rice cultivars assessed, the variation in three chosen traits represented the typical ranges documented for rice products: head rice recovery percentage: HRR%, *T*_*3*_) ∈ **[**45% to 73%] similarly as documented in [36], (where HRR% ranged from 24 to 74%), the chalky rice kernel percentage *T*_2_ (CRK%) spanning from *T*_2_ ∈ **[**1% to 90%] (similar to [37], where the CRK% ranged from 1 to 75%), and chaffy grain counts *T*_1_ ∈ **[**0% to 100%]. It took ~ 6 min to evaluate ~ 5 g of rice grains for all three traits. Since the accuracies achieved using the analysis from X-ray images were in reasonable agreement with the manual reference methods these might justify the next steps towards robustification of these methods and might be, potentially, considered for the rice grain evaluation in the future.

Particularly, the ability to predict several grain traits from single X-ray images (in our case HRR%, CRK%, chaffiness) on an individual grain level could be of immense value to users. Moreover, there is an opportunity to develop further algorithms calculating additional traits from these images (e.g. size and shape, weight, grain matter density as demonstrated before [11]). This imaging approach can, in theory, substantially streamline the sample-handling logistics and minimize the sample-manipulation related errors. The non-destructive nature of X-ray image-based evaluation, furthermore, opens the opportunity to re-use the same samples for other tests/sowing. Already in sugar beet quality testing and some sorting applications X-ray CT or X-ray radiography methods are used regularly without further effects on germination behavior [38]. In the presented method here, each grain receives a dose of approximately $\text{3,12}\cdot {10}^{-3}$ Gy (HRR% and CRK%) and $\text{0,51}\cdot {10}^{-3}$ Gy (Chaffiness). Thus, it is not likely to affect the germination behavior of individual grains. Nevertheless, it is reported that a reduction in germination behavior is after accumulated doses of more than 15 Gy [39] and plant growth is affected at doses above 30 Gy [39], both being between 5000 and 30000 times higher than the dose applied within the current experiment.

### Novel opportunities

We demonstrated it is feasible to develop algorithms to infer relatively complex structural-physical traits from paddy rice based on 2D X-ray projections (HRR%, chaffiness, CRK%). This image analysis included several processing steps enabling the detection, the segmentation, and the evaluation of single rice grains even for the bulk trait as HRR% where ground-truth cannot be measured on individual grains. The individual grain analysis opened the opportunity to assess rice cultivars more accurately or select individual grains for further testing.

For the case of CRK%, we showcased that the raw 2D X-ray images of whole rice grains can be virtually segmented into kernels and husk (“virtually de-husked”) and the kernel-features can be reliably evaluated from the whole grain scans. The ability of X-ray system to evaluate internal structures of grains without milling process can be considered another substantial advantage for logistics of grain evaluation, particularly for the tightly husked grains (such as rice, barley, small millets, sunflower, safflower etc.).

### Limitations and further directions

The presented study demonstrated the X-ray based imaging system could be used to evaluate rice grains, yet further developments are required to achieve the level of technological robustness to integrate such methodology into routine operations. Next step of system development will be guided by close collaboration with the system users. Ultimately, robustification of the trait inference algorithms by adding more grain ground truth measurements from diverse rice cultivars with their choice being guided by the users’ requirements will have to be done. Also, in the current state of the algorithm development, the “virtual de-husking algorithms” requires further standardization for particular types of paddy rice (see Sect. “Rice grain material and ground truth measurements”). Furthermore, the sample holders hand-crafted for the study should be standardized and improved to ease the grain handling operations. Other development steps might include transition from 2D radiography to 3D tomography, which might ramp up the size of the sample being evaluated at one go (nevertheless, 3D imaging procedures will have to be carefully optimized as 3D tomographic methods are more time-intensive in terms of scanning and require high computation intensity). Recent literature also illustrated that the grains and kernels can be evaluated directly in the panicle without threshing [24, 40] which could be the next step to ease logistics of the sample preparations, especially for those crops where threshing/shelling pose a hurdle for grain evaluation (such as rice, small millets, barley etc.). Nowadays, it's becoming feasible to engineer robust, portable, X-ray systems to evaluate the paddy grains/panicles outdoors [41, 42]. Technology mobilization might propel the usage of the above demonstrated research beyond the current infrastructural limitations (e.g. for paddy evaluation directly in the commodity value-chains) and is in the spotlight of global Food & Safety organizations [43].

## Conclusion

The presented proof-of-concept study demonstrated, for the first time, that a single X-ray image of the paddy rice grains coupled with adequate trait prediction models can be used to evaluate multiple physical and structural grain and kernel characteristics (e.g. traits such as HRR%, chaffiness, CRK%). The presented non-destructive X-ray image analysis returns trait values for individual rice grains and doesn’t require grain milling to evaluate grain kernel characteristics. Therefore, the presented method might significantly enhance accuracy and efficiency of rice grain evaluations thus enhance rice research. These principles can be readily adapted for other grain crops and are expected to enhance the effectivity of grain evaluation processes, particular for the tightly husked grains (e.g. barley, small millets, sunflower) where high throughput is expected.

With the expected trends for miniaturization and mobilization of technology we foresee the integration of the proposed X-ray technology with many other research areas (e.g. commodity value chains, climate- change agricultural transition).

## Data Availability

No datasets were generated or analysed during the current study.

## Abbreviations

CT: Computed tomography
HRR%: Head rice recovery percentage
CRK%: Chalky rice kernel percentage
PCA: Principal component analysis
SVM: Support vector machine
FOD: Focus object distance
FDD: Focus detector distance
RMSE: Root mean squared error

## Footnotes

1 https://www.iis.fraunhofer.de/en/ff/zfp/products/ctportable.html

2 https://www.iis.fraunhofer.de/en/f/zfp/products/volex10
